# Supplementary material for: Causal Relationship between Aging and Anorexia Nervosa: A White-Matter-Microstructure-Mediated Mendelian Randomization Analysis
Source: Biomedicines. 2024 Aug 16;12(8):1874. doi: 10.3390/biomedicines12081874 (PMC11351342; doi:10.3390/biomedicines12081874)
Supplement: Supplementary file 1 [file biomedicines-12-01874-s001.zip › Supplementary Figure/Figure S4/Singe SNP plot of MR analysis for TL to ubm-b-1688.pdf]

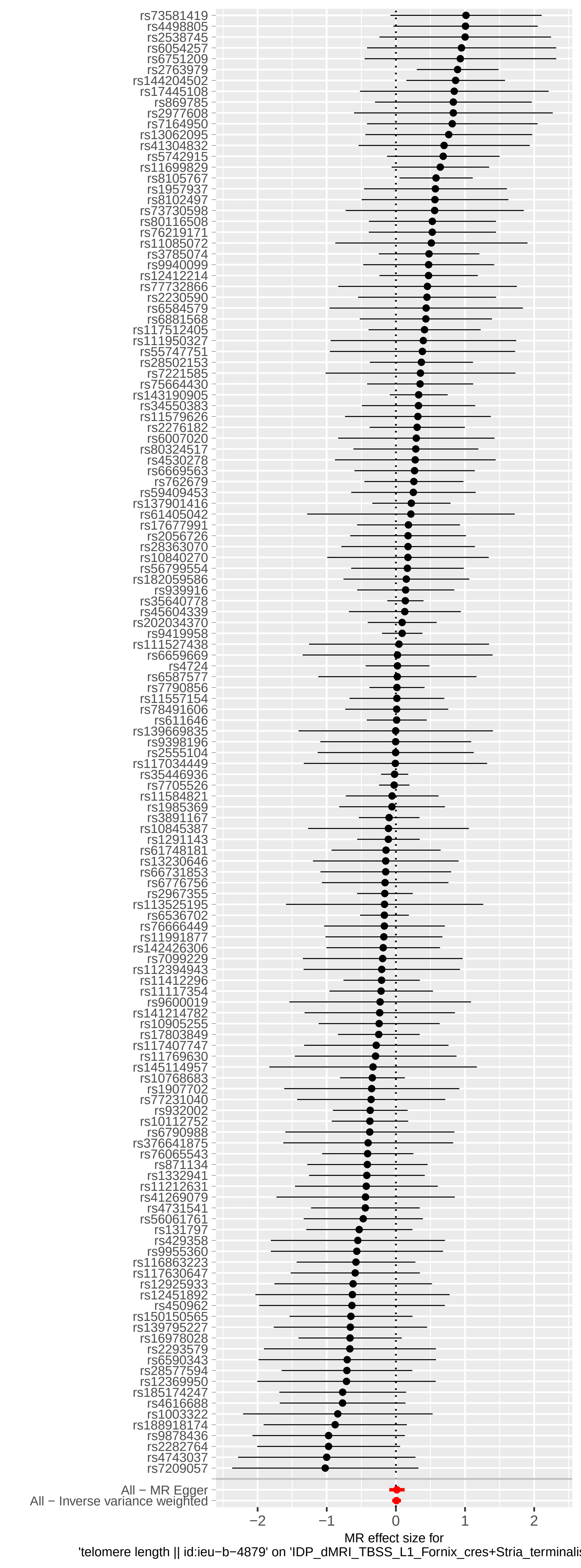

rs73581419
rs4498805
rs2538745
rs6054257
rs6751209
rs2763979
rs144204502
rs17445108
rs869785
rs2977608
rs7164950
rs13062095
rs41304832
rs5742915
rs11699829
rs8105767
rs1957937
rs8102497
rs73730598
rs80116508
rs76219171
rs11085072
rs3785074
rs9940099
rs12412214
rs77732866
rs2230590
rs6584579
rs6881568
rs117512405
rs111950327
rs55747751
rs28502153
rs7221585
rs75664430
rs143190905
rs34550383
rs11579626
rs2276182
rs6007020
rs80324517
rs4530278
rs6669563
rs762679
rs59409453
rs137901416
rs61405042
rs17677991
rs2056726
rs28363070
rs10840270
rs56799554
rs182059586
rs939916
rs35640778
rs45604339
rs202034370
rs9419958
rs111527438
rs6659669
rs4724
rs6587577
rs7790856
rs11557154
rs78491606
rs611646
rs139669835
rs9398196
rs2555104
rs117034449
rs35446936
rs7705526
rs11584821
rs1985369
rs3891167
rs10845387
rs1291143
rs61748181
rs13230646
rs66731853
rs6776756
rs2967355
rs113525195
rs6536702
rs76666449
rs11991877
rs142426306
rs7099229
rs112394943
rs11412296
rs11117354
rs9600019
rs141214782
rs10905255
rs17803849
rs117407747
rs11769630
rs145114957
rs10768683
rs1907702
rs77231040
rs932002
rs10112752
rs6790988
rs376641875
rs76065543
rs871134
rs1332941
rs11212631
rs41269079
rs4731541
rs56061761
rs131797
rs429358
rs9955360
rs116863223
rs117630647
rs12925933
rs12451892
rs450962
rs150150565
rs139795227
rs16978028
rs2293579
rs6590343
rs28577594
rs12369950
rs185174247
rs4616688
rs1003322
rs188918174
rs9878436
rs2282764
rs4743037
rs7209057
All − MR Egger
All − Inverse variance weighted
−2
−1
0
1
2
MR effect size for
'telomere length || id:ieu−b−4879' on 'IDP_dMRI_TBSS_L1_Fornix_cres+Stria_terminalis
